# A novel autophagy-related gene signature associated with prognosis and immune microenvironment in ovarian cancer

**DOI:** 10.1186/s13048-023-01167-5

**Published:** 2023-04-29

**Authors:** Jiani Yang, Chao Wang, Yue Zhang, Shanshan Cheng, Meixuan Wu, Sijia Gu, Shilin Xu, Yongsong Wu, Yu Wang

**Affiliations:** 1 Department of Gynecology, Shanghai First Maternity and Infant Hospital, School of Medicine, Tongji University, Shanghai, 200092 China; 2 Shanghai Key Laboratory of Maternal Fetal Medicine, Shanghai First Maternity and Infant Hospital, School of Medicine, Shanghai Institute of Maternal-Fetal Medicine and Gynecologic Oncology, Tongji University, Shanghai, 200092 China; 3 Department of Obstetrics and Gynecology, Renji Hospital, School of Medicine, Shanghai Jiaotong University, Shanghai, China

**Keywords:** Autophagy, Ovarian cancer, Prognostic signature, FOXO1, Tumor immune microenvironment

## Abstract

Ovarian cancer (OV), the most fatal gynecological malignance worldwide, has high recurrence rates and poor prognosis. Recently, emerging evidence supports that autophagy, a highly regulated multi-step self-digestive process, plays an essential role in OV progression. Accordingly, we filtered 52 potential autophagy-related genes (ATGs) among the 6197 differentially expressed genes (DEGs) identified in TCGA-OV samples (*n* = 372) and normal controls (*n* = 180). Based on the LASSO-Cox analysis, we distinguished a 2-gene prognostic signature, namely FOXO1 and CASP8, with promising prognostic value (*p*-value < 0.001). Together with corresponding clinical features, we constructed a nomogram model for 1-year, 2-year, and 3-year survival, which was validated in both in training (TCGA-OV, *p*-value < 0.001) and validation (ICGC-OV, p-value = 0.030) cohorts. Interestingly, we evaluated the immune infiltration landscape through the CIBERSORT algorithm, which indicated the upregulation of 5 immune cells, including CD8 + T cells, Tregs, and Macrophages M2, and high expression of critical immune checkpoints (CTLA4, HAVCR2, PDCD1LG2, and TIGIT) in high-risk group. Stepwise, high-risk group exhibited better sensitivity towards chemotherapies of Bleomycin, Sorafenib, Veliparib, and Vinblastine, though less sensitive to immunotherapy. Especially, based on the IHC of tissue microarrays among 125 patients in our institution, we demonstrated that aberrant upregulation of FOXO1 in OV was related to metastasis and poor prognosis. Moreover, FOXO1 could significantly promote tumor invasiveness, migration, and proliferation in OV cell lines, which was assessed through the Transwell, wound-healing, and CCK-8 assay, respectively. Briefly, the autophagy-related signature was a reliable tool to evaluate immune responses and predict prognosis in the realm of OV precision medicine.

**Supplementary Information:**

The online version contains supplementary material available at 10.1186/s13048-023-01167-5.

## Introduction

Ovarian cancer (OV) is the most fatal gynecological malignancy worldwide, with high recurrence rates and poor prognosis, which seriously threatens women’s safety and health [1]. As reported in the United States, there were 12,810 deaths and 19,880 new cases of OV, estimated for 2022 [2]. Due to the lack of typical symptoms, over 70% OV cases were diagnosed at advanced stage, leading to the poor 5-year overall survival (OS) rate of only 35%, regardless of recent advances in OV treatments [3, 4]. Given the poor prognosis, studies exploring the underlying mechanism of OV metastasis are urgently needed to improve survival.

Autophagy is a highly regulated multi-step self-digestive process via which cells adapt to stress conditions [5]. During the autophagy process, cytoplasmic materials, including organelles and macromolecules, are engulfed in autophagosomes, the specialized double-membrane structure which could fuse with lysosomes to form autolysosomes for cargos degradation and nutrients regeneration [6, 7]. Under normal conditions, autophagy is kept at a basal level for housekeeping purposes, including turnover of damaged cellular organelles and degradation of long-lived proteins. In response to diverse stimuli like oxidative reagents and serum starvation, autophagy is induced for cellular metabolism maintenance, thus facilitating cell survival [7]. Accumulating evidence shows that autophagy plays an essential role in starvation adaptation, cell cycle regulation, and cancer, including OV [6, 8]. However, since the role of autophagy is either related to cell death or cyto-protection, the specific mechanism that directly links the autophagic process and cancer progression need clarification.

The mammalian Forkhead Box protein (FOXOs) family, including FOXO1, FOXO3, FOXO4, and FOXO6, is essential in various intra-cellular functions, including cell cycle, apoptosis, and autophagy [9]. Among them, FOXO1, a gene located on human chromosome 13q4, is one of the most widely studied members [10]. Post-translational modification, especially acetylation of FOXO1, is a vital mechanism involved in defense against oxidative stress, DNA repair, apoptosis, and cell cycle arrest [11, 12]. Researchers have also reported that FOXO1 could regulate the autophagy mediated by curcumin and benzyl isothiocyanate [13], though haven’t been verified in OV yet.

Accordingly, in our study, we comprehensively evaluated the importance of ATGs in OV, and filtered FOXO1 and CASP8 to identify the prognostic signature. Moreover, we assessed the tumor immune microenvironment and sensitivity to chemotherapy/immunotherapy between risk-groups stratified by the signature. We aimed to investigate the vital role of autophagy, especially through FOXO1 in OV metastasis, as a reliable tool to evaluate immune responses and affect patient survival.

## Methods

### Patients and specimens

Primary ovarian cancer samples (*n* = 125) and metastatic samples (*n* = 40) were obtained from 125 OV patients, who underwent cytoreductive surgery, followed by standard platinum-based chemotherapy, in Renji Hospital between June 2007 and December 2013. Another 38 cases of normal fallopian tube or ovarian tissues were obtained as controls. Clinicopathological data were obtained from medical records. Follow-up visits were performed every 3 months for the first 2 years, every 6 months for the next 3 years and then annually through clinical or radiological evaluation. Each patient was followed until January 2022. OS was identified from the date of surgery to the last follow-up visit or death. Progression-free survival (PFS) was measured from the date of surgery to the last follow-up visit or cancer progression, which was assessed by radiographic and clinical evidence. This research was approved by the Ethics Committee of Renji Hospital Affiliated to Shanghai Jiaotong University School of Medicine and all patients provided informed consents for the usage of their information and samples for research purposes.

### Data collection and autophagy-related genes filtration

**Fig. 1:**
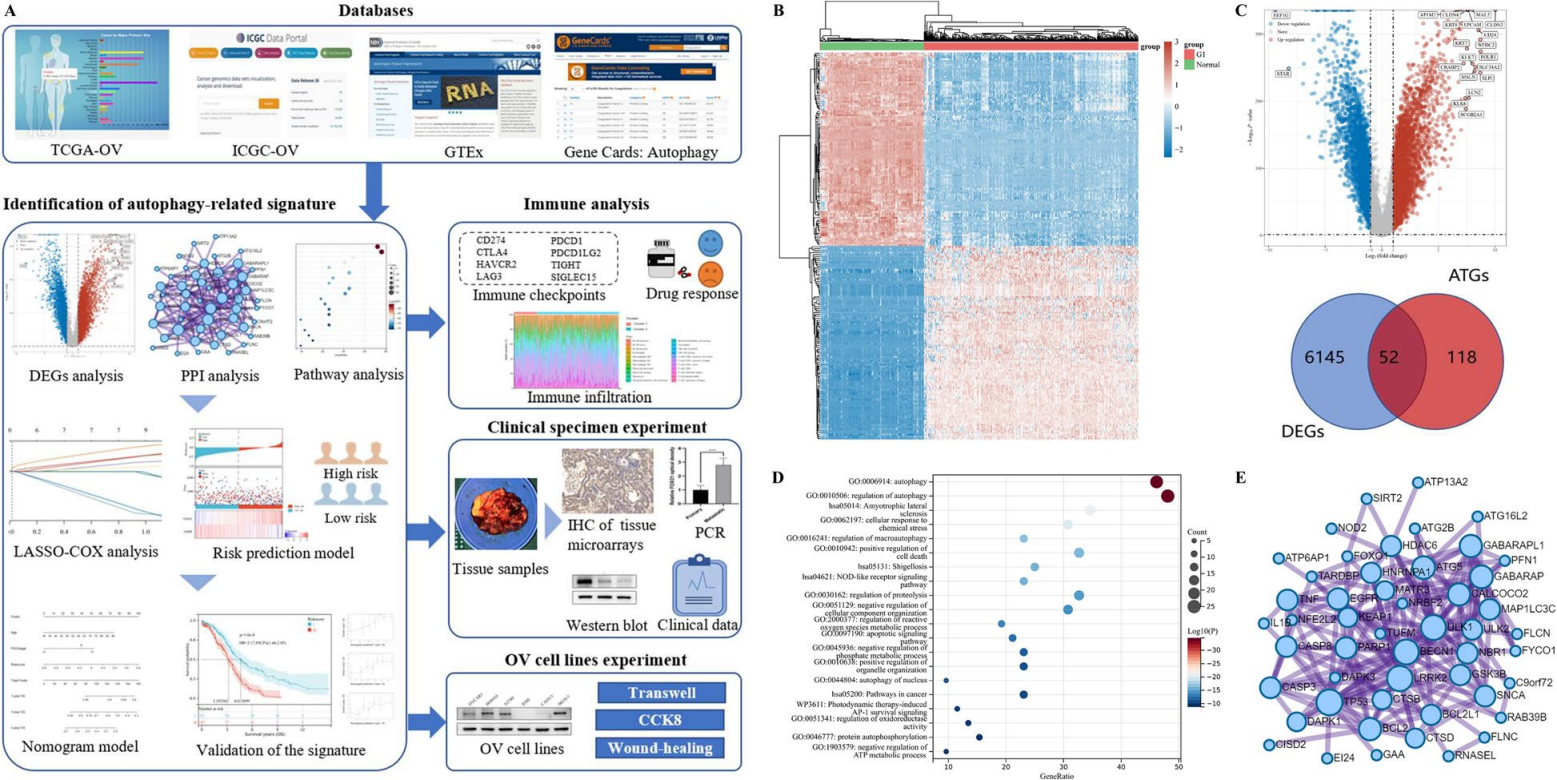
Identification of differentially expressed autophagy-related genes (DE-ATGs) in ovarian cancer (OV). **A** The flowchart of the study. **B** The heatmap of differential-expressed genes (DEGs) in normal and OV tissues of The Cancer Genome Atlas Ovarian Cancer (TCGA-OV) dataset, among which the top 50 down-regulated genes and top 50 up-regulated genes were illustrated. **C** The volcano plot (top) of 6197 DEGs was constructed refer to adjust p-value and fold change values. The red dots indicate up-regulated genes, while the blue dots indicate downregulated genes (|log2(FC)|> 1 and adjust *p*-value < 0.01). The Venn diagram (bottom) showed the 52 significant differentially expressed autophagy-related genes (DE-ATGs). **D** The Kyoto Encyclopedia of Genes and Genomes (KEGG) and Gene Ontology (GO) pathway enrichment analysis were performed to explore the functions of potential DE-ATGs. Here, the top 20 clusters were listed. **E** The protein–protein interaction (PPI) network diagram of the 52 DE-ATGs

Figure 1A graphed the workflow of the study. Autophagy-associated genes (relevance score > 4) were retrieved at the GeneCards website (https://www.genecards.org/) by searching the term “autophagy.” Both RNA-sequencing (RNA-seq) datasets and corresponding clinical characteristics of OV patients were downloaded at the Cancer Genome Atlas database (TCGA; https://portal.gdc.com) as the training set, and the International Cancer Genome Consortium database (ICGC; https://dcc.icgc.org) as the validation set. The volcano plot was constructed refer to the fold change values and adjust *p*-value, while the heatmap of the differential gene expression was graphed with top 50 up-regulated and 50 down-regulated genes. Moreover, to confirm underlying functions of potential genes, we annotated the targets by Gene Ontology (GO) and the Kyoto Encyclopedia of Genes and Genomes (KEGG) analysis.

### Construction and validation of the autophagy-related prognostic signature

The least absolute shrinkage and selection operator (LASSO) Regression algorithm with tenfold cross-validation was adopted to filter potential ATGs for signature construction. Subsequently, we conducted both univariate and multivariate Cox Regression analysis to define ATGs for the prognostic signature. The linear combination method was performed to assemble expression level and coefficient of each selected ATGs to obtain a risk score formula as following: risk-score = ∑β * Exp, where β is the regression coefficient of each prognostic gene, and Exp is the expression level of it. The “glmnet” package of R software was used to identify the autophagy-related prognostic signature and calculate the risk score of each patient based on the signature. The samples were then divided into low-risk and high-risk groups based on the medium risk score as the cutoff value.

To assess the prognostic value of the signature, we graphed the Kaplan–Meier (K-M) curves, which were stratified by the medium cut-off value of risk-score. Next, we also conducted the time-dependent Receiver Operating Characteristic (ROC) analysis via the “timeROC” package. Both univariate and multivariable Cox Regression analyses were performed to access indicators related to OS. Then, we build the nomogram based on the identified factors to predict 1-year, 3-year, and 5-year OS, with a graphical representation.

### Tumor immune microenvironment analysis and drug sensitivity assessment

In order to evaluate the immune infiltration landscape, we analyzed the composition of the 22 typical immune cells infiltrating in the tumor microenvironment of OV samples based on the CIBERSORT algorithm (https://cibersortx.stanford.edu/) [14]. To identify effective immunotherapy for OV patients, we assessed immune checkpoint gene expression (including CTLA4, CD274, HAVCR2, LAG3, PDCD1LG2, PDCD1, TIGIT, and SIGLEC15). Additionally, in order to predict the potential Immune Checkpoint Blockade (ICB) response for OV patients, we downloaded RNA-sequencing expression profiles and corresponding clinical data from the TCGA database (https://portal.gdc.com). Then, potential ICB response for OV patients was predicted with the Tumor Immune Dysfunction and Exclusion (TIDE) algorithm (http://tide.dfci.harvard.edu.), a computational framework developed by Jiang and colleagues to model the tumor immune escape and predict ICB response [15]. To predict patient response to chemotherapy, we also evaluated the Half-maximal Inhibitory Concentration Values (IC50) based on the Genomics of Drug Sensitivity in Cancer database (GDSC, https://www.cancerrxgene.org/).

### Patients and specimens

Primary ovarian cancer samples (*n* = 125) and metastatic samples (*n* = 40) were obtained from 125 OV patients in Renji Hospital between June 2007 and December 2013. All the patients involved have received optimal debulking surgery, followed by 6-cycle standard platinum-based chemotherapy. Another 38 cases of normal fallopian tube or ovarian tissues were obtained as controls. Clinicopathological data were obtained from medical records. Follow-up visits were performed every 3 months for the first 2 years, every 6 months for the next 3 years and then annually through clinical or radiological evaluation. Each patient was followed until January 2022. OS was identified from the date of surgery to the last follow-up visit or death. Progression-free survival (PFS) was measured from the date of surgery to the last follow-up visit or cancer progression, which was assessed by radiographic and clinical evidence. This research was approved by the Ethics Committee of Renji Hospital Affiliated to Shanghai Jiaotong University School of Medicine and all patients provided informed consents for the usage of their information and samples for research purposes.

### Cell lines

The human OV cell lines (including A2780, SKOV3, ES-2, HO-8910, and OVCAR-3), IOSE cell line, and embryonic kidney cell line 293 T were obtained from the American Type Culture Collection (ATCC, Manassas, VA). HO-8910, SKOV3, and OVCAR-3 cells were maintained in RPMI 1640 (Gibco, USA), while A2780, ES-2, 293 T, and IOSE were maintained in Dulbecco’s modified Eagle’s medium (Gibco, USA). To make a complete growth medium, we add 10% fetal bovine serum (Gibco, GranIsland, USA) and 100 mg/mL of penicillin and streptomycin (Invitrogen). The cell lines were mycoplasma free and kept in an incubator with saturated humidity and 5% CO^2^ at 37 °C. All the cell lines were regularly authenticated based on the recommendations of the ATCC cell bank via short tandem repeat polymorphism analysis.

### Immunohistochemistry evaluation

For the immunohistochemistry (IHC) assay, samples were de-waxed, followed by hydration and wash. After the microwave antigen retrieval process, the sections were treated with 3% H^2^O^2^ for blockage of endogenous peroxidase activity. Then, slides were incubated overnight with Anti-FOXO1 antibody (Servicebio, GB11286-1, 1:100). Subsequently, the slides were incubated by horseradish peroxidase (HRP)-conjugated secondary antibody (Abclonal, AS014) and related signals were visualized by diaminobenzidine and counter-stained by hematoxylin.

Two experienced pathologists scored IHC signal intensity and percentage independently, without prior information about the samples. Histochemistry score (H-score) was determined based on the intensity of nuclear staining and the proportion of labeled tumor cells: H-Score = ∑(pi × i) = percentage of weak intensity cells × 1 + percentage of moderate intensity cells × 2 + percentage of strong intensity cells × 3. The staining intensity was graded from 0 to 3 (0 = negative, 1 = weak, 2 = moderate and 3 = strong). The final H-score ranges from 0 to 300, in which higher score was defined as higher expression [16].

### RNA isolation and quantitative real-time PCR

Total RNA from cells was extracted via Trizol Reagent (Merk, T9424) and reverse transcribed to Cdna using the RevertAid First Strand Cdna Synthesis Kit (Thermo Fisher Scientific, K1622) following the manufacturer’s instructions. Then, the real-time quantitative reverse transcription-polymerase chain reaction (RT-PCR) analysis was performed using SYBR Green Master Mix (Thermo Fisher Scientific, A25742) on an QuantStudio™ 7 Flex Real-Time PCR System (Applied Biosystems by Life technologies, USA). All RT-PCR reactions were repeated at least three times and conducted in triplicates. Primer sequences were designed as follows: GAPDH, Forward: 5′- GGCAAATTCCATGGCACCG-3′ and Reverse: 5′- TCGCCCCACTTGATTTTGGA-3′; FOXO1, Forward: 5′-TCAGGTGGTGGAGATCGACC-3′ and Reverse: 5′- CCGAGTTGGACTGGCTAAACTC-3′. The comparative expression level was evaluated by 2-ΔΔCt method, while GAPDH was set as an internal control.

### Western blot analysis

Total protein of cells was extracted through ice-cold radioimmunoprecipitation assay (RIPA) lysis buffer (Thermo Fisher Scientific, 89,900), containing the protease inhibitor cocktail (Merk, P8340). Then, the proteins were quantified by BCA Protein Assay Kit (Beyotime, P0010) and boiled for degeneration. Subsequently, we separated proteins in SDS-PAGE (Beyotime, P0012A) and transferred them on the PVDF membrane (Merk, 3,010,040,001). After being blocked in 5% Bovine serum albumin (BSA, Solarbio, SW3015), membranes were incubated with primary antibodies: Anti-FOXO1 (Abclonal, A2934, 1:1000) and Anti-beta-actin (Proteintech, 20,536, 1:1000). Then, the membranes were incubated in secondary antibodies: Goat Anti-Mouse IgG (Proteintech, SA00001-1, 1:1000) and Goat Anti-Rabbit IgG (Proteintech, SA00001-2, 1:1000), followed by enhanced chemiluminescence to display bands.

### Plasmids and cell transfection

Lentiviral vector Ubi-MCS-3FLAG-CBh-gcGFP-IRES-puromycin was purchased from Shanghai Genechem Company. The FOXO1 Cdna was cloned downstream of the lentiviral vector by homologous recombination. Lentiviral vector FOXO1 was purchased from the Cdna Library of School of Medicine, Shanghai Jiaotong University. Based on the manufacturer’s instruction, plasmids were transfected into cells by using the LipoHigh transfection reagent (Sangon Biotech, E607403) in serum-free Opti-MEM (Thermo Fisher Scientific, 31,985,070). An empty vector was used as a negative control. Then, cells were screened by puromycin (Sangon Biotech, A610593).

### Transwell assay, wound-healing assay, and CCK-8 assay

For the Transwell invasion assay, OV cells (approximately 1 × 10^5^ per well) were cultured with 200 μL of serum-free medium and seeded in the upper chamber of each insert (8-μm pore size, Corning, 3422), which was pre-coated with Matrigel (Corning, 354,234). Meanwhile, the bottom chambers were filled with 600 μL of medium, containing 10% FBS. After incubation at 37 °C for 72 h, cells unable to pass through the membrane were erased, whereas those invaded through the membrane were fixed with 4% paraformaldehyde solution (Sangon Biotech, E672002), stained by crystal violet solution (Servicebio, G1014).

We used the Cell Counting Kit-8 (CCK-8, Dojindo, CK04) assay to evaluate the proliferation ability of OV cells, refer to the manufacturer’s instruction. 100 μL per well of treated cells were inoculated in 96-well plates at the density of 2 × 10^4^cells / ml for 24,48 and 72 h, respectively. At each indicated time point, 10 μL of CCK-8 solution per well was added and incubated for 2 h. Then, the absorbance value (450 nm) was analyzed through a microplate reader (BioTek, Synergy H1). The experiment was repeated at least three times.

### Statistical analysis

Quantitative data was expressed as mean ± standard deviation (SD) and analyzed by T-test. Category data was expressed as numbers and percentages and compared through the χ2 test. Univariate and multivariate analyses of clinicopathological characteristics were conducted through the Cox hazards regression model. Statistical analyses were performed using the R version 4.0.3 (foundation for statistical computing 2020) and graphed through the Graph Prism Software (Version 7.0a, GraphPad). For all tests, *p*-value < 0.05 was defined as statistically significant.

## Results

### Identification of differentially expressed autophagy-related genes in OV

Firstly, we downloaded the gene expression profiles of the TCGA-OV cohort (*n* = 372) with corresponding clinical characteristics and prognosis information. Moreover, we also involved the GTEx cohort (*n *= 180) as controls (Fig. 1B). Following the analysis of the TCGA-OV dataset, a total of 6197 differential-expressed genes (DEGs) were identified in OV and normal samples, which were shown by the volcano plot (Fig. 1C, top). Then, we obtained a total of 170 autophagy-related genes (ATGs) with relevance score > 4 at the GeneCards database. As displayed in the Venn diagram, the 52 significant differentially expressed autophagy-related genes (DE-ATGs) were selected for further analysis (Fig. 1C, bottom). In addition, to explore the underlying functions of DE-ATGs, we performed pathway enrichment analysis via the Metascape website (https://metascape.org) [17]. In Fig. 1D, we listed the top 20 most significant KEGG and GO pathways, which were mainly enriched in regulation of autophagy, cellular response to chemical stress, regulation of cell death, pathways in cancer, etc. Based on the Search Tool for Retrieval of Interacting Genes database (https://string-db.org), we conducted a protein–protein interaction (PPI) network among the 52 DE-ATGs, so as to reveal protein interactions, which might provide some hints for further exploration of the underlying mechanism (Fig. 1E) [18].

### Construction and estimation of a prognostic signature based on the ATGs

**Fig. 2:**
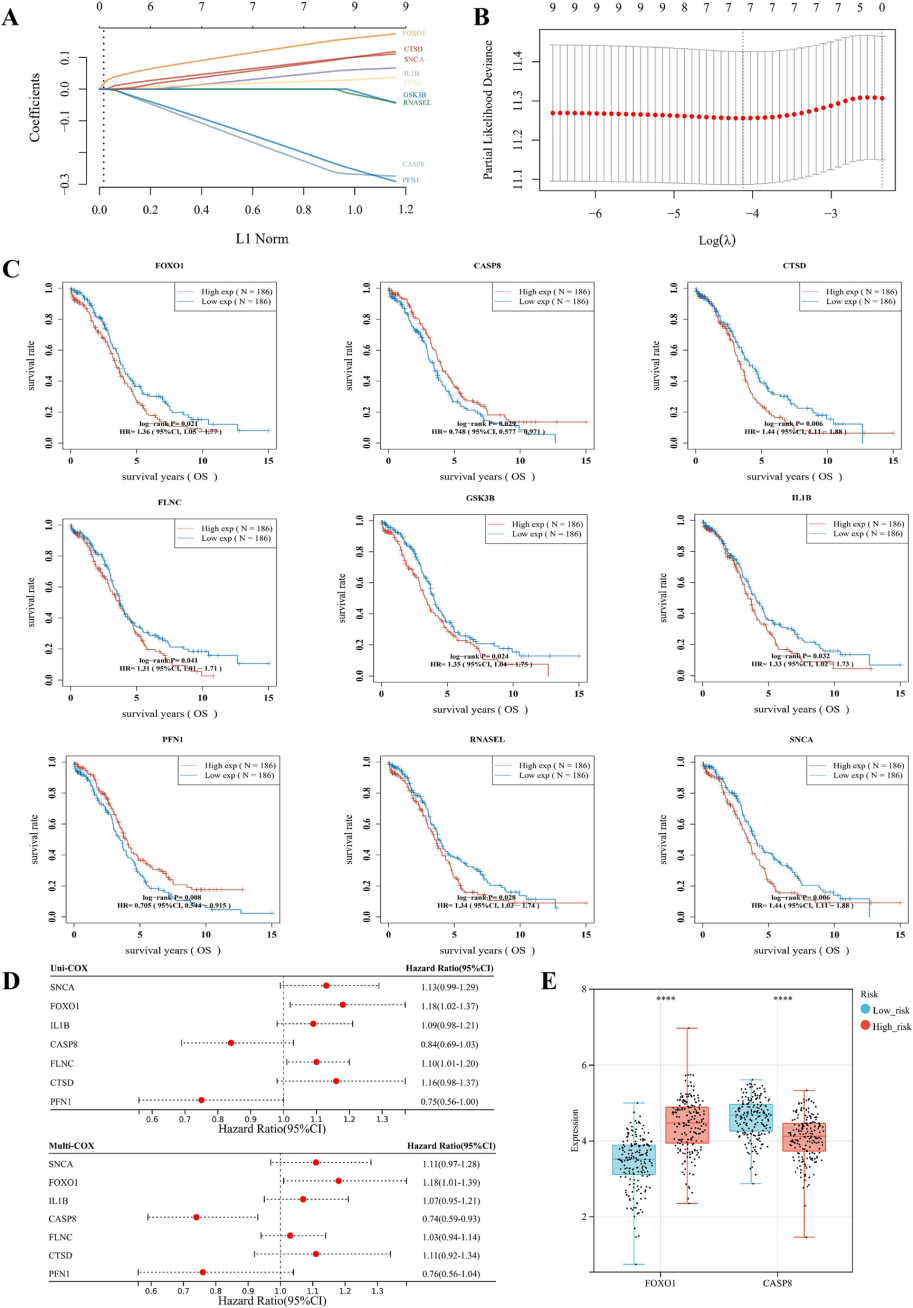
Construction of an ovarian cancer (OV) prognostic signature based on the autophagy-related genes (ATGs). **A** The λ selection diagram for the LASSO tuning parameter selection, with tenfold cross-validation. **B** The LASSO-Cox analysis for 9 optimal prognostic ATGs, including FOXO1, CASP8, CTSD, FLNC, GSK3B, IL1B, PEN1, RNASEL, and SNCA. **C** The Kaplan–Meier (K-M) curves of the 9 potential prognostic ATGs. **D** The forest diagrams indicated the prognostic value of the 9 potential ATGs, which were analyzed through univariate and multivariate Cox Regression algorithms. **E** The expression distribution of 2 selected prognostic PRGs, namely FOXO1 and CASP8, in normal tissues and OV tissues

Through the LASSO regression analysis, 9 potential prognostic genes were filtered (including FOXO1, CASP8, CTSD, FLNC, GSK3B, IL1B, PEN1, RNASEL, and SNCA) from the 52 DE-ATG (Fig. 2A and B). Based on the Kaplan–Meier (K-M) survival curves, the 9 DE-ATGs were significantly associated with the OS of TCGA-OV (Fig. 2C). OV patients with high expression of CASP8 and PEN1 had better OS, while those with high expression of FOXO1, CTSD, FLNC, GSK3B, IL1B, RNASEL, and SNCA suffered worse OS. The overview for functions of the 9 potential DE-ATGs with prognostic value in OV was listed in Table 1. To enhance explicability, both univariate and multivariate Cox Regression analysis was conducted, which distinguished 2 prognostic genes for the prognostic signature, namely FOXO1 and CASP8 (Fig. 2D). Ultimately, the autophagy-related 2-gene prognostic signature was evaluated through the multivariate Cox Regression analysis as follows: risk-score = (0.218) * FOXO1 + (-0.2374) * CASP8. The expression distribution of FOXO1 and CASP8 in normal tissues and OV tissues is presented in Fig. 2E, among which FOXO1 was up-regulated, and CASP8 was downregulated in OV.

**Table 1:** The overview for functions of the nine differentially expressed autophagy-related genes (DE-ATGs) with prognosis value in ovarian cancer (OV). [19–28]

Gene	Gene name	Functions in OV	Reference
FOXO1	Forkhead Box protein 1	FOXO1 is essential in various intra-cellular functions, including autophagy. The phosphorylation of FOXO1 regulated by ITGA2 could regulate resistance to paclitaxel in OV	[19]
CASP8	Caspase 8	The genetic variants of CASP8 could protect against carcinogenesis and delay tumor onset in OV. CASP8 inhibition could regulate cancer progression by triggering the autophagy process based on ATG3 and BECLIN-1	[20, 21]
CTSD	Cathepsin D	The overexpression of CTSD in OV tumor tissue could enhanced proangiogenic responses including angiogenic tube formation, proliferation, and migration via activation of the PI3K/AKT and ERK1/2 pathways	[22, 23]
GSK3B	Glycogen synthase kinase 3 beta	The activation of ERK/GSK3β/β-catenin pathway by lipocalin2 could promote tumor cell proliferation and migration in OV	[24]
IL1B	Interleukin 1 beta	In OV, IL1B could regulate the NF-κB pathway to induce the up-regulation of HLA-G expression, which is correlated with microenvironment tolerant immune cells, such as Tregs and the diminution of memory T and NK cells	[25]
FLNC	Filamin C	Unknown in OV. In gastric cancer, FLNC downregulation by acetylated Siah2 could increase invasiveness of tumor cells	[26]
PEN1	Penetration 1	Unknown in OV	-
RNASEL	Recombinant Ribonuclease L	Unknown in OV. As for prostate cancer, RNASEL could mediate the proapoptotic activities of the IFN-inducible 2-5A system, which is important in prostate cancer susceptibility	[27]
SNCA	Synuclein alpha	Unknown in OV. SNCA, a small cytoplasmic protein that involves in neurodegenerative diseases, is expressed in a high percentage of OVs	[28]

**Fig. 3:**
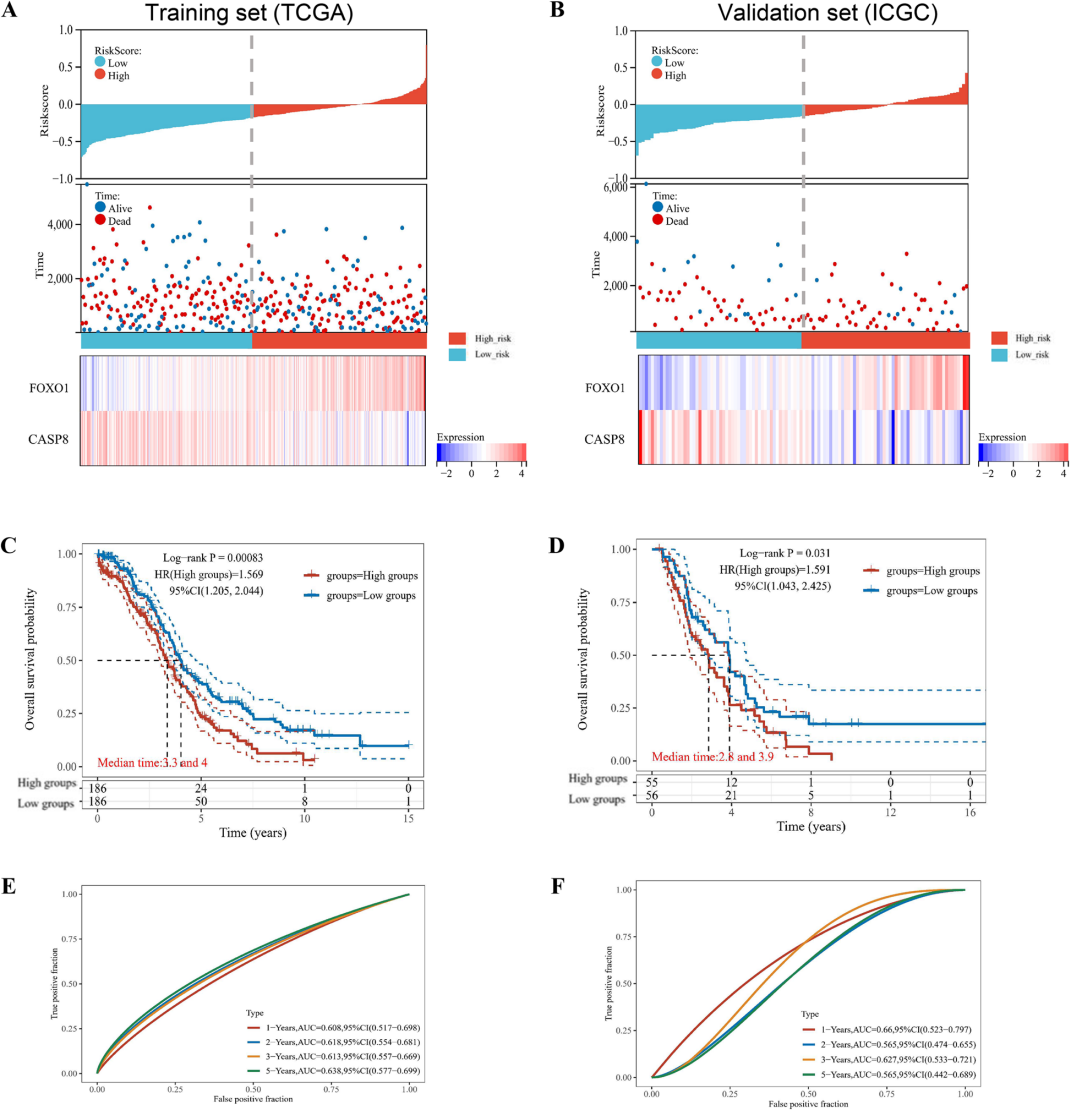
Validation and estimation of the ovarian cancer (OV) prognostic signature based on the autophagy-related genes (ATGs). The distribution of risk-scores, survival status and survival time (months) of ovarian cancer (OV) individuals among the (**A**) TCGA-OV training set and (**B**) ICGC-OV validation set. The scatter plots showed risk-scores of OV patients, refer to corresponding survival status and survival (top and middle). The heatmaps (bottom) represented the expression profiles of the gene signature of FOXO1 and CASP8 in two risk groups. The Kaplan–Meier (K-M) overall survival curves for OV patients of the (**C**) TCGA-OV training set and (**D**) ICGC-OV validation set, which were classified into low-risk and high-risk groups. The ROC analysis for the OS prediction value of the autophagy-related signature, among both the (**E**) TCGA-OV training set and (**F**) ICGC-OV validation set

Stepwise, we calculated the risk-score for OV patients, including the training cohort (TCGA-OV set; *n* = 372) and the validation set (ICGC-OV set; *n* = 111), based on the above formula. Refer to the median cut-off value, we stratified OV patients into two risk groups: low-risk and high-risk. We illustrated the risk-scores of OV patients in the training and validation cohorts (Fig. 3A and B, respectively), referring to corresponding status and survival time (top and middle). We also listed the expression profiles of the signature in two groups. In both training and validation cohorts, FOXO1 was highly expressed in the high-risk group, while CASP8 was highly expressed in the low-risk group. The K-M analysis indicated that low-risk OV patients had better OS in both the training cohort (*p*-value < 0.001) and validation cohort (*p*-value = 0.031) (Fig. 3C and D, respectively). Then, we performed the time-dependent ROC analysis, which demonstrated that the autophagy-related signature had promising prognostic values for 1-year, 3-year, and 5-year OS in both the training cohort (Fig. 3E) and validation cohort (Fig. 3F).

### Construction and validation of an autophagy-related prognostic nomogram

Stepwise, we evaluated the relationship between the autophagy-related signature and clinical characteristics, including age, race, grade, and FIGO stage (Supplement Fig. 1A-D, respectively), which indicated no significance (*p*-value ≥ 0.05). The Sankey diagram visualized the distribution of each OV patient, based on the autophagy-related signature and corresponding clinical features, including age, pathological grade, and the FIGO stage ( Supplement Fig. 1E).

**Fig. 4:**
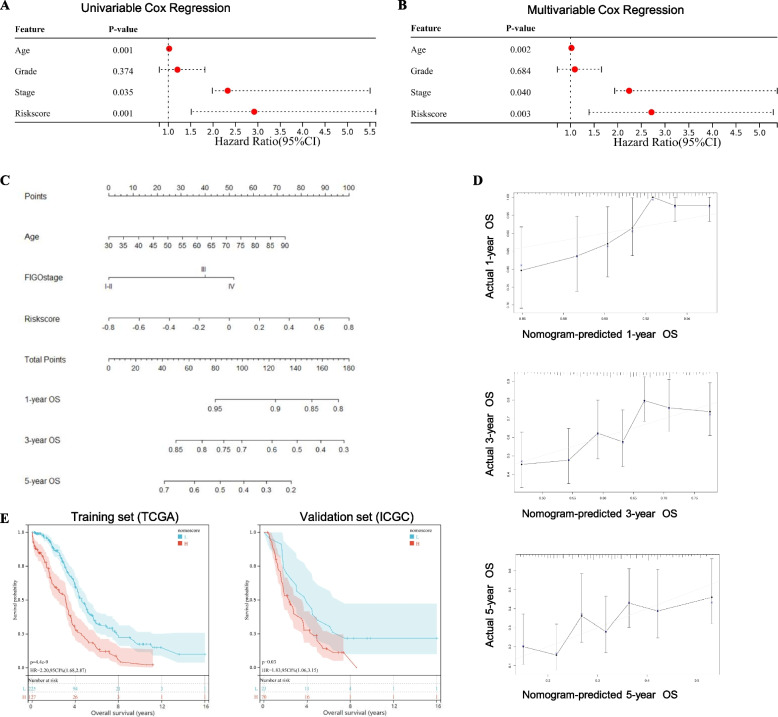
Construction and validation for the autophagy-related 2-gene-based prognostic nomogram. The forest plots presented the (**A**) univariate and (**B**) multivariate Cox Hazard Regression analysis for ovarian cancer (OV) patient survival, based on the autophagy-related 2-gene signature and clinical features (including age, FIGO stage, and grade). **C** The prognostic nomogram model was conducted to predict the 1-year, 3-year, and 5-year overall survival (OS) of OV patients, based on the autophagy-related signature and clinical indicators selected by the Cox Hazard Regression analysis (namely age and FIGO stage). **D** The calibration curves for the prognosis nomogram model for predicting 1-year (top), 3-year (middle), and 5-year (bottom) OS. The diagonal line represented the ideal nomogram, while the black lines represented the observed nomogram. **E** The Kaplan–Meier (K-M) curves for OV patients in the training cohort (TCGA-OV) and validation cohort (ICGC-OV), which were stratified according to the nomogram score

In order to distinguish prognostic indicators for OV patients, we conducted both univariable and multivariable Cox Regression analyses, which indicated that age (*p*-value = 0.002), clinical FIGO stage (*p*-value = 0.040), and risk-score (*p*-value = 0.003) were independent factors for OV prognosis (Fig. 4A and B). Based on the integration of these indicators, we constructed a prognostic nomogram model to predict the 1-year, 3-year, and 5-year overall survival (OS) of OV patients (Fig. 4C). The concordance index (C-index) of the nomogram with and without the risk-score was 0.635 and 0.590, respectively. The calibration plots showed agreement between actual observation and nomogram prediction, in terms of the 1-year, 3-year, and 5-year survival rate, suggesting appreciable reliability of the nomogram model (Fig. 4D). Based on the above model, we calculated the nomogram score for every OV patient, which were further stratified into two groups, based on the median cut-off value. Then, the K-M survival curves demonstrated that OV patients with high nomogram scores suffered a worse prognosis in both training cohort (TCGA-OV, *p*-value < 0.001) and validation cohort (ICGC-OV, *p*-value = 0.030) (Fig. 4E).

### Immunity analysis of the tumor immune microenvironment based on the autophagy-related signature

**Fig. 5:**
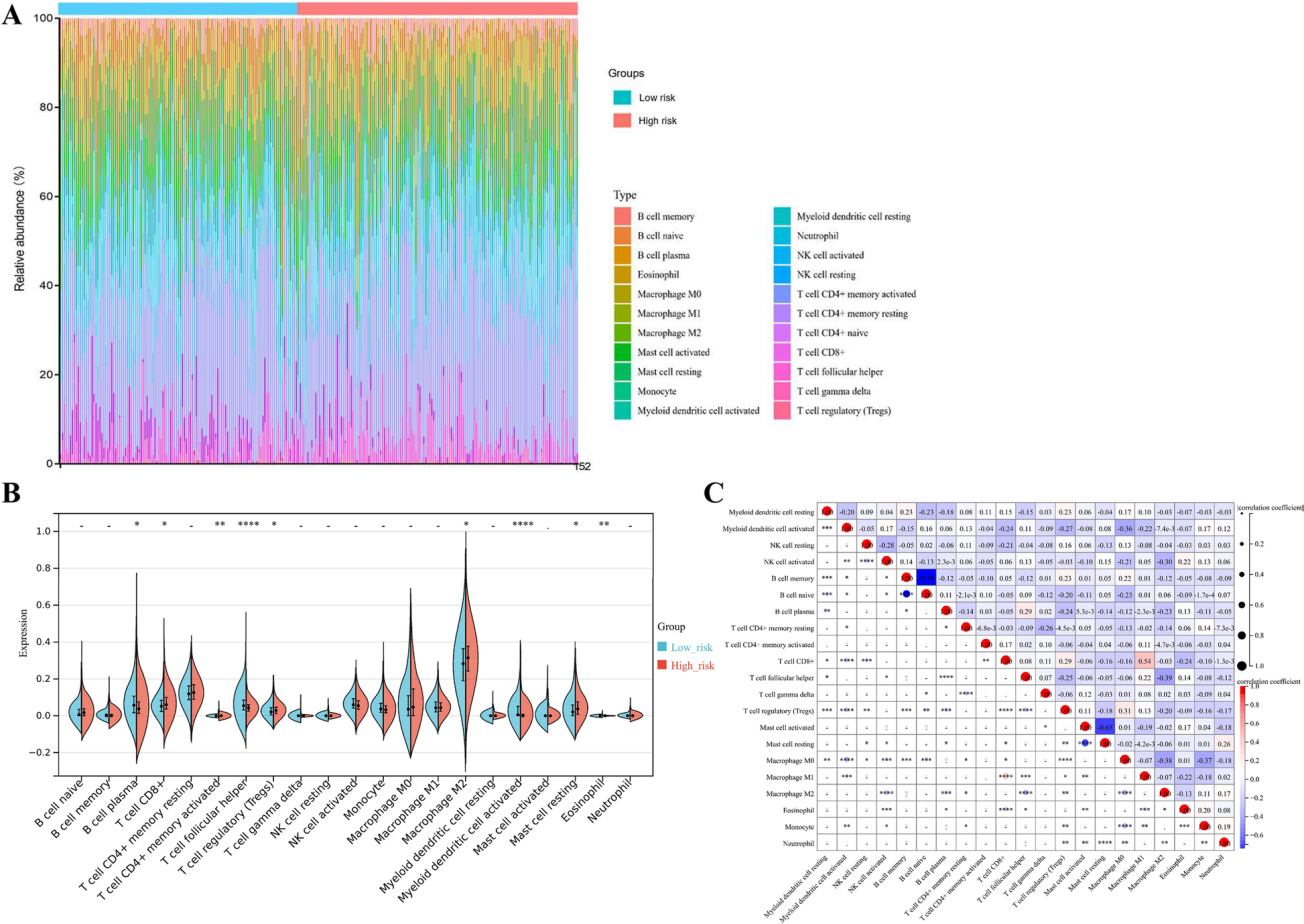
The immunity analysis for the tumor immune microenvironment based on the autophagy-related signature. **A** The Boxplots represented composition of the 22 typical immune cells infiltrating in the tumor microenvironment of OV samples in both low-risk and high-risk groups, based on the CIBERSORT algorithm. **B** The Violin plots showed the distribution of the 22 typical immune cells infiltration in two risk groups, which were stratified by the autophagy-related signature. **C** The heatmaps represented the relationships of the 22 typical immune cells infiltrating in OV samples. *p-value < 0.05; **p-value < 0.01; ***p-value < 0.001; ****p-value < 0.0001

Nowadays, emerging studies focus on the cross-talk between tumor cells and immune cells, which suggests that the tumor immune microenvironment could play a vital role in OV progression [29]. Accordingly, we evaluated the immune infiltration landscape of OV patients classified by the autophagy-related signature, in order to assess the association between autophagy and tumor immune microenvironment. We summarized the composition of the 22 typical immune cells infiltrating in the tumor microenvironment of OV samples in both low-risk and high-risk groups, based on the CIBERSORT algorithm (Fig. 5A). According to the analysis, 5 out of the 22 typical immune cells, including CD8 + T cells, activated CD4 + memory T cells, regulatory T cells (Tregs), Macrophages M2, and resting mast cells were significantly up-regulated in the high-risk group, while plasma B cells, follicular helper T cells, activated Myeloid Dendritic Cells (DCs), and eosinophils were down-regulated (Fig. 5B). Except for inherent relationships between activated and corresponding resting immune cells, Macrophages M2 and follicular helper T cells had the strongest negative association (correlation coefficient = -0.39; *p*-value < 0.0001), while Macrophages M1 and CD8 + T cells had the strongest positive association (correlation coefficient = 0.54; *p*-value < 0.0001) (Fig. 5C).

### Assessment of OV patient response to chemotherapy and immunotherapy

**Fig. 6:**
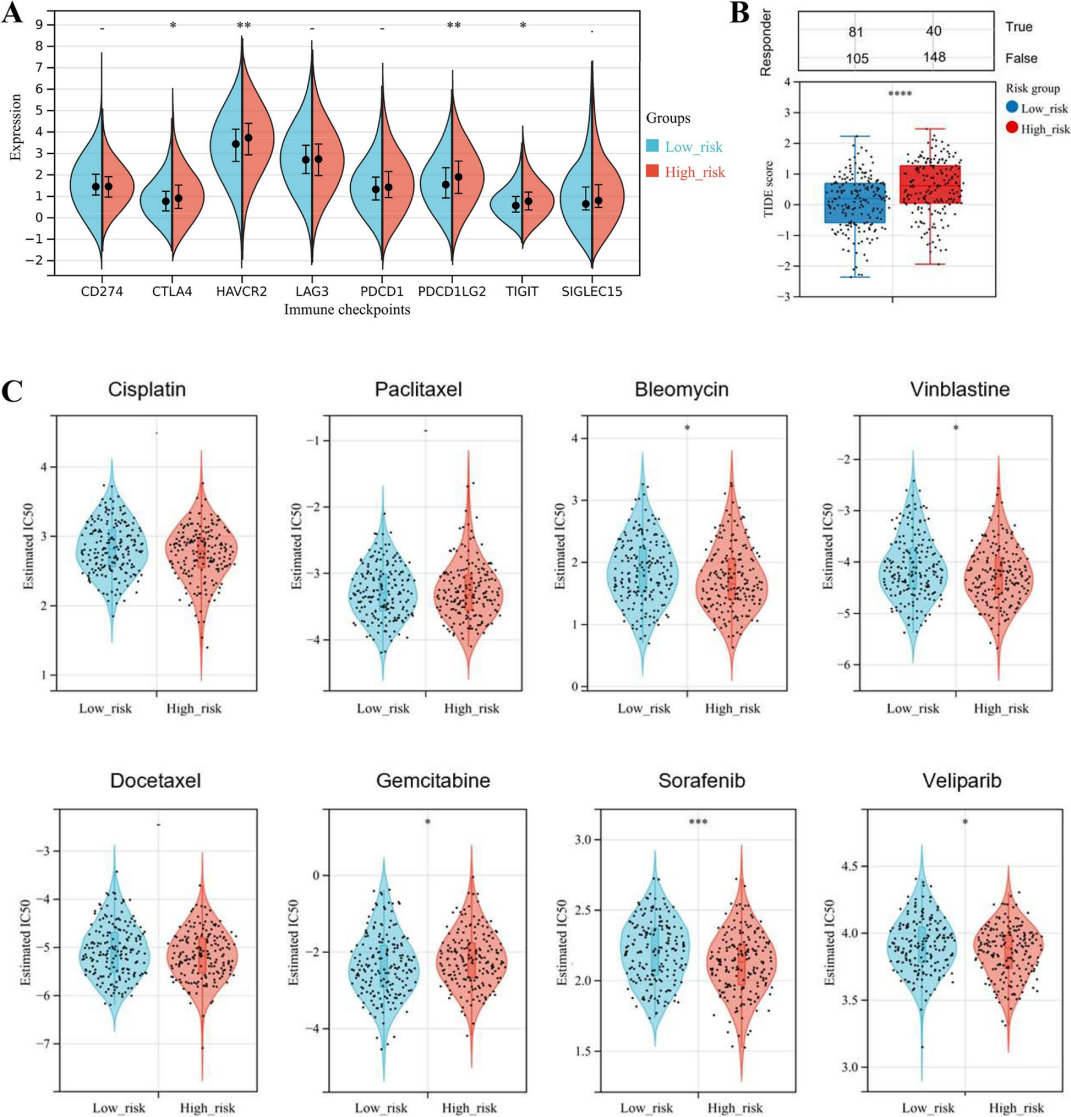
Assessment of OV patient sensitivity to immunotherapy and chemotherapy. **A** The boxplots showed the expression difference of eight typical immune checkpoints (including CTLA4, CD274, HAVCR2, LAG3, PDCD1LG2, PDCD1, TIGIT, and SIGLEC15) between the two risk groups stratified by the autophagy-related signature. **B** Based on the Tumor Immune Dysfunction and Exclusion (TIDE) scoring system, we evaluated the sensitivity of OV patients towards immunotherapy. **C** The violin plots represented the estimated half-maximal inhibitory concentration (IC50) values of OV patients towards 8 typical chemotherapies, including Cisplatin, Paclitaxel, Bleomycin, Docetaxel, Gemcitabine, Sorafenib, Veliparib, and Vinblastine. The chemotherapy sensitivity analyses were carried out through the Genomics of Drug Sensitivity in Cancer database (GDSC). *p-value < 0.05; **p-value < 0.01; ***p-value < 0.001; ****p-value < 0.0001

Additionally, according to the RNA-sequencing expression (level 3) profiles of the TCGA-OV cohort, we evaluated the association between immune checkpoint expression and the autophagy-related signature, which showed that CTLA4, HAVCR2, PDCD1LG2, and TIGIT were significantly up-regulated in the high-risk group (*p*-value < 0.05, Fig. 6A). Thus, OV patients in the high-risk group were more likely to benefit from those immunotherapies, especially focusing on the immune checkpoints of CTLA4, HAVCR2, PDCD1LG2, and TIGIT. Based on the RNA-sequencing expression profiles of the TCGA-OV cohort, we predicted the response of OV patients towards immune checkpoint blockade (ICB) through the TIDE algorithm [15], a computational framework that could model tumor immune escape and predict ICB response (Fig. 6B). The results implied that OV patients in the high-risk group had higher TIDE scores, which represented worse efficacy and poorer prognosis after the ICB treatment (*p*-value < 0.05).

In order to evaluate the chemotherapy response of two risk groups, we assessed the estimated half-maximal inhibitory concentration (IC50) values of 8 typical chemotherapies, including Cisplatin, Paclitaxel, Bleomycin, Docetaxel, Gemcitabine, Sorafenib, Veliparib, and Vinblastine through the GDSC database (https://www.cancerrxgene.org/), the largest publicly available pharmacogenomics database. The results demonstrated that in the high-risk group, the estimated IC50 values of Bleomycin, Sorafenib, Veliparib, and Vinblastine were significantly lower, while the estimated IC50 of Gemcitabine was higher. Accordingly, OV patients with high risk-score were more sensitive to Bleomycin, Sorafenib, Veliparib, and Vinblastine, though less sensitive to Gemcitabine. No significant difference was found between the two risk groups, refer to the sensitivity to Cisplatin, Paclitaxel, and Docetaxel (*p*-value ≥ 0.05) (Fig. 6C).

### Aberrant upregulation of FOXO1 in OV was related with metastasis and poor prognosis

**Fig. 7:**
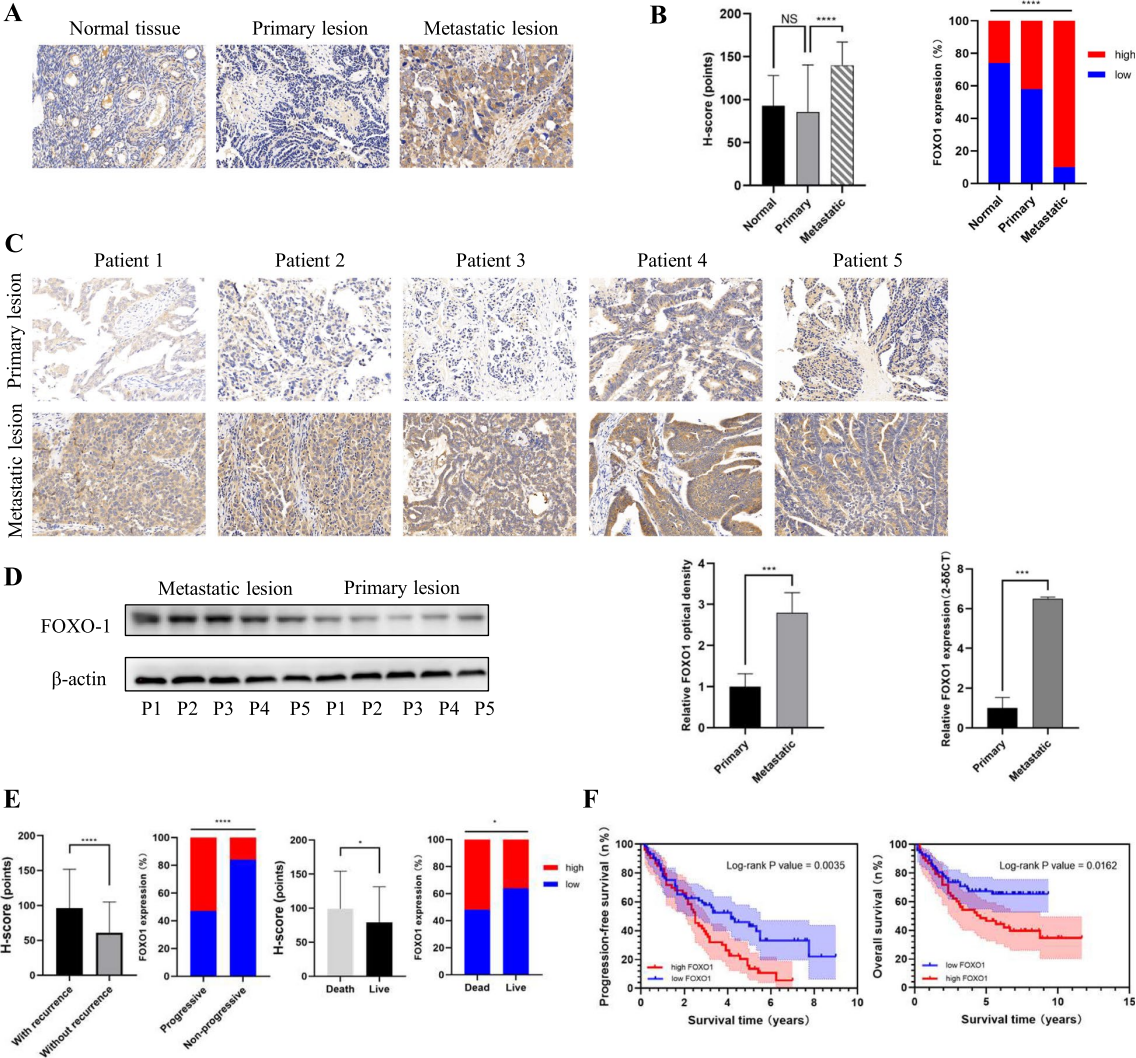
FOXO1 expression is up-regulated in OV and relates to poor clinical outcomes. **A** Representative immunohistochemistry (IHC) staining images of FOXO1 expression of various specimens (including the normal ovary tissue, primary OV lesion, and metastatic lesion) were shown. Original magnification × 200. **B** Compared with normal ovary tissues and primary OV lesions, metastatic lesions had higher FOXO1 expression, measured by the Histochemistry score (H-score). **C** Representative IHC staining images of primary and metastatic lesions from 5 OV patients. Original magnification × 200. **D** Western blotting (left) and PCR analysis (right) of FOXO1 protein and mRNA expression in primary and metastatic lesions from 5 OV patients. **E** Analyzed through IHC staining, FOXO1 expression was increased in patients who suffered progression or death. **F** Upregulation of FOXO1 correlates with poor survival. Kaplan–Meier survival curves for the progression-free survival (PFS, left) and overall survival (OS, right) of 125 OV patients were shown, which were divided into subgroups based on FOXO1 expression

IHC of tissue microarrays indicated that FOXO1 expression staining was mainly located at the cytoplasm of tumor cells (Fig. 7A). Compared with normal ovary tissues and primary OV lesions, metastatic lesions had significantly higher FOXO1 expression, which was measured through the mean H-score of 92.78 ± 35.49, 85.82 ± 54.62, and 140.08 ± 26.99, respectively (Fig. 7B). Moreover, the percentages of samples with high FOXO1 expression (H-score ≥ 100) increased gradually in three groups: normal ovary tissues (26.32%, 10/38), primary OV lesions (42.40%, 53/125), and metastatic lesions (90.00%, 36/40) (*p*-value < 0.05) (Fig. 7B). Representative IHC staining images of primary and metastatic lesions from 5 OV patients were listed in Fig. 7C. Stepwise, Western Blotting and PCR analysis of the samples indicated that FOXO1 expression was increased in metastatic lesions, at both protein and mRNA levels (Fig. 7D). Through IHC staining analysis of the 125 OV cases, FOXO1 expression was increased in patients who suffered progression or death, compared with the survivors (Fig. 7E).

In addition, the association between FOXO1 expression and clinicopathological characteristics of all OV patients was shown in Table 2, with no significant differences among various FOXO1 expression groups refer to age, FIGO stage, pathology stage, histology type, tumor diameter, and CA-125 (*p*-value ≥ 0.05). The median OS and PFS for all patients were 34 months (range 14–52) and 59 months (range 25–89), respectively. Refer to the K-M survival curves, FOXO1 expression was significantly associated with both PFS (*p*-value = 0.004, Fig. 7F, left) and OS (*p*-value = 0.016, Fig. 7F, right) in OV patients. To further determine independent prognostic factors, we performed both univariate and multivariate analyses (Table 3). The results revealed that FIGO stage (HR 3.780; 95% CI, 1.787–7.996; *p*-value = 0.001) and FOXO1 expression (HR 1.796; 95% CI, 1.023–3.152; *p*-value = 0.041) were significantly associated with OV prognosis. Collectively, these results strongly implied that FOXO1 was correlated with OV metastasis and poor prognosis.

**Table 2:** The correlation between FOXO1 expression and clinicopathological characteristics of 125 OV patients

Characteristic	FOXO1 expression		*p*-value
	**Low (H-score < 100)**	**High (H-score ≥ 100)**	
**Age (n,%)**			0.318
** < 55 years**	35(62.5%)	21(37.5%)	-
** ≥ 55 years**	37(53.6%)	32(46.4%)	-
**FIGO stage (n,%)**			0.246
**I-II**	29(64.4%)	16(35.6%)	-
**III-IV**	43(53.8%)	37(46.2%)	-
**Pathology stage (n,%)**			0.155
**I-II**	35(64.8%)	19(35.2%)	-
**III**	37(52.1%)	34(47.9%)	-
**Histology type (n,%)**			0.062
**Serous**	51(65.4%)	27(34.6%)	-
**Mucous**	6(4.8%)	5(4.0%)	-
**Endometrioid**	4(28.6%)	10(71.4%)	-
**Other types**	11(50.0%)	11(50.0%)	-
**Tumor diameter (n,%)**			0.179
** < 10 cm**	32(51.6%)	30(48.4%)	-
** ≥ 10 cm**	40(63.5%)	23(36.5%)	-
**Serum CA125 (n, %)**			0.813
** < 35 U/ml**	12(60.0%)	8(40.0%)	-
** ≥ 35 U/ml**	60(57.1%)	45(42.9%)	-

*Abbreviation*: *H-score* Histochemistry score, *FIGO stage* Federation of International of Gynecologists and Obstetricians stage

**Table 3:** Univariate and multivariate analysis of prognostic factors among 125 OV patients

Characteristic	Univariate Analysis		Multivariate Analysis	
	**HR (95% CI)**	***P*-value**	**HR (95% CI)**	***P*-value**
**Age**				
** < 55 years**	Reference	-	Reference	-
** ≥ 55 years**	0.937(0.552–1.591)	0.81	0.802(0.462–1.392)	0.432
**FIGO stage**				
**I-II**	Reference	-	Reference	-
**III-IV**	3.042(1.570–5.895)	0.001	3.780(1.787–7.996)	0.001
**Pathology stage**				
**I-II**	Reference	-	Reference	-
**III**	0.767(0.454–1.296)	0.322	0.710(0.393–1.284)	0.257
**Histology type**		0.829		0.193
**Serous**	Reference	-	Reference	-
**Mucous**	1.450(0.641–3.280)	0.372	3.001(0.976–9.221)	0.055
**Endometrioid**	0.980(0.410–2.341)	0.963	0.990(0.397–2.471)	0.983
**Other types**	1.143(0.563–2.321)	0.711	1.686(0.765–3.716)	0.195
**Tumor diameter**				
** < 10 cm**	Reference	-	Reference	-
** ≥ 10 cm**	1.300(0.768–2.199)	0.329	1.312(0.739–2.328)	0.353
**Serum CA125**				
** < 35 U/ml**	Reference	-	Reference	-
** ≥ 35 U/ml**	1.656(0.749–3.660)	0.213	1.459(0.538–3.954)	0.458
**FOXO1 expression**				
**Low (H-score < 100)**	Reference	-	Reference	-
**High (H-score ≥ 100)**	1.892(1.113–3.214)	0.018	1.796(1.023–3.152)	0.041

*Abbreviation*: *HR* hazard ratio, *95% CI* 95% confidence interval, *FIGO stage* Federation of International of Gynecologists and Obstetricians stage, *H-score* Histochemistry score

### FOXO1 promotes tumor metastasis in OV cell lines

**Fig. 8:**
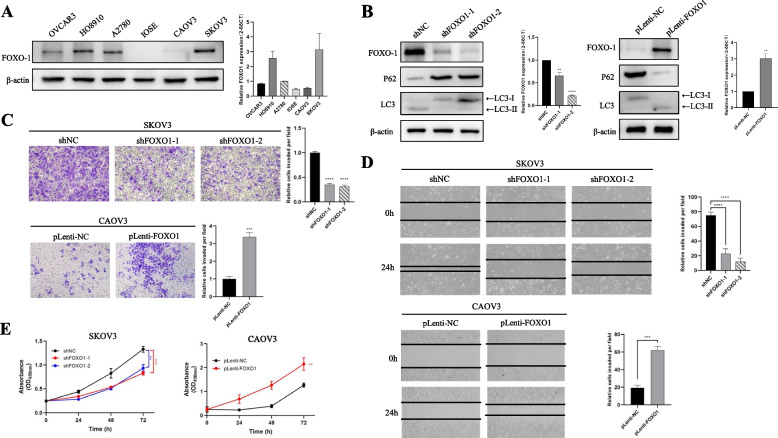
FOXO1 promoted OV cell metastasis in vitro. **A** Western blotting (left) and PCR (right) analysis of FOXO1 protein and mRNA expression in IOSE and OV cell lines, including OVCAR3, HO8900, A2780, CAOV3, and SKOV3. **B** Western blotting was performed to evaluate the FOXO1 expression, p62 degradation and, LC3-II accumulation in SKOV3 cells when FOXO1 is knockdown, in response to serum starvation (SS) for 12 h. PCR verification of FOXO1 knockdown was also conducted (left). Western blotting was conducted to determine the FOXO1 expression, p62 degradation and, LC3-II accumulation in CAOV3 cells when FOXO1 is overexpressed, in response to SS. PCR verification of FOXO1 overexpression was also performed (right). **C** The invasiveness of SKOV3 cells was suppressed by the knockdown of FOXO1, while the invasiveness of CAOV3 cells was promoted by overexpression of FOXO1. Original magnification × 100. **D** The migration of SKOV3 cells was suppressed by the knockdown of FOXO1, while the migration of CAOV3 cells was promoted by overexpression of FOXO1. The wound-healing process was monitored for 24 h and observed under microscopy. Original magnification × 40. **E** Measured by the Cell Counting Kit-8 (CCK-8) assay, FOXO1 promoted the proliferation of SKOV3 and CAOV3 cells. The error bar represents the mean ± standard deviation (SD). **p-value < 0.01; ***p-value < 0.001; ****p-value < 0.0001

To explore the role of FOXO1 in tumorigenesis and progression of OV, we compared the levels of FOXO1 expression in IOSE and OV cell lines, including OVCAR3, HO8900, A2780, CAOV3, and SKOV3, through western blot and qRT-PCR. Among all OV cell lines included, the protein and mRNA expression level of FOXO1 was highly-expressed in SKOV3 and A2780 cells, meanwhile lowly expressed in CAOV3 cells (Fig. 8A). Then, lentivirus-mediated knockdown of FOXO1 expression in SKOV3 cells and overexpression of FOXO1 in CAOV3 cells were conducted to establish stable infectants (Fig. 8B). In addition, based on the autophagy flux assays published by Klionsky and colleagues [30], the FOXO1 levels were significantly related to the autophagic process, in that LC3-II accumulation and p62 degradation were decreased in SKOV3 cells with FOXO1 knockdown (Fig. 8B, left), while increased in CAOV3 cells with FOXO1 overexpression (Fig. 8B, right). The Transwell chamber migration experiment confirmed that knockdown of FOXO1 significantly inhibited cell invasiveness in SKOV3 cell lines; in contrast, overexpression of FOXO1 promoted cell invasiveness in CAOV3 lines (Fig. 8C). The wound-healing test showed that the migration ability of SKOV3 cells was suppressed by FOXO1 knockdown, while the migration of CAOV3 cells was promoted by FOXO1 overexpression (Fig. 8D). Measured by the CCK-8, we proved that FOXO1 overexpression could promote the proliferation of SKOV3 and CAOV3 cells (Fig. 8E). These results indicated that FOXO1 significantly promoted OV cell metastasis in vivo. Moreover, we also evaluated the levels of autophagy activity change when FOXO1 is knockdown, based on the autophagy flux assays published by Klionsky and colleagues [30].

## Discussion

OV was the most fatal gynecological malignance worldwide, mainly due to high recurrence rate and lack of sufficient biomarkers [1, 31]. Therefore, identifying a promising signature and exploring the underlying mechanism of OV metastasis is of great urgency. Recently, emerging evidence supported that autophagy, a type II programmed cell death regulated by a multi-step self-digestive process, played crucial roles in cancer progression, by providing survival advantages to the OV cells that face metabolic stress and protecting them from organelles and macromolecules damage induced by tumor therapy [6, 7]. Therefore, in our research, we comprehensively evaluated the importance of ATGs in OV and identified the prognostic signature (namely FOXO1 and CASP8), which was related to tumor immune microenvironment and sensitivity to immunotherapy/ chemotherapy. Moreover, we aimed to explore the vital role of ATGs, especially FOXO1, in OV metastasis, in order to present a new therapeutic target and assist clinical decision-making in the future.

Up till now, though several comprehensive studies have focused on the relationship between autophagy and OV prognosis, none of the previous autophagy-related signatures have been applied to clinical practice yet, partly due to limitations in sensitivity and specificity. Chen and colleagues constructed and validated an autophagy-related 7-gene signature for OV prognosis [32]. However, only 4 out of the 7 ATGs played significant prognostic roles in OV, including ATG12, GABARAPL1, ULK2, and IFNG, while ATG4A, ATG4C, and ATG5 were not statistically significant (*p*-value ≥ 0.05). Another study by Fei and colleagues identified another prognostic signature, which consisted of 5 ATGs, including CXCR4, DNAJB9, HSP90AB1, PEX3, and RB1, without external validation [33]. Accordingly, we tried to distinguish a reliable autophagy-related signature from 170 potential ATGs downloaded from the Genecards dataset. Then, we identified a 2-gene signature (including FOXO1 and CASP8), which was validated in both training (TCGA-OV, *p*-value < 0.001) and validation (ICGC-OV, *p*-value = 0.030) cohorts. To the best of our knowledge, our research is initial to define the autophagy-related signature of FOXO1 and CASP8, which had satisfactory prognostic value for OV patients.

Nowadays, the cross-talk between tumor cells and immune cells has gained increasing attention, along with increasing breakthroughs in immune checkpoint inhibitors [34]. Therefore, we assessed the landscape of immune infiltration in OV, through the CIBERSORT analysis. In the high-risk group, 5 out of the 22 typical immune cells, including Macrophages M2, CD8 + T cells, activated CD4 + memory T cells, Tregs, and resting mast cells were significantly up-regulated, while plasma B cells, follicular helper T cells, activated DCs, and eosinophils were downregulated. Baek and colleagues performed molecular images of OV cells expressing Enhanced Firefly Luciferase (Effluc) in living mice and demonstrated that Macrophages M2 could accelerate OV progression [35]. Moreover, consistent with our findings, Foucher et al. implied that M-CSF-induced macrophages could switch memory T cells into Th17 cells through membrane IL-1α, which was required in OV metastasis [36]. As for activated DCs, Lee and colleagues concluded that activated DCs played a vital role in immune responses in the process of OV progression, in regards of T cell recruitment into tissue, activated memory T cells maintenance, and T cell response initiation [37, 38]. However, the immune landscapes still need further validation and exploration of the underlying mechanism.

During the past decades, regardless of the advances in anti-tumor therapies, clinical treatments for OV still face the bottleneck of the 80% recurrence rate, which might due to limited sensitivity towards immunotherapy and chemotherapy [31, 39]. Emerging evidence revealed that autophagy, a catabolic process degrading intracellular components of lysosomes, was a bridge to tumor immunity, which could influence patient sensitivity to anti-tumor therapies [40, 41]. Accordingly, our study explored the relationship between autophagy patterns and sensitivity to anti-tumor therapies. Based on the GDSC dataset, we indicated that high risk-score OV patients were more sensitive to Bleomycin, Sorafenib, Veliparib, and Vinblastine, though less sensitive to Gemcitabine. In addition, high-risk patients had higher TIDE score, which represented worse efficacy and poorer prognosis after the ICB treatment. Interestingly, the high-risk group was more likely to benefit from those immunotherapies of the immune checkpoints, including CTLA4, HAVCR2, PDCD1LG2, and TIGIT. Our findings hinted that the underlying mechanism of immune checkpoint therapy in OV should be more complicated than directly targeting the immune checkpoints. However, further research is still needed to improve the accuracy of cell line-based predictors of patient response to immunotherapies and chemotherapies.

There were limited researches that reported definite functions of the identified ATGs (namely FOXO1 and CASP8) in OV progression. Previous studies concluded that CASP8 inhibition was enough to trigger the autophagy process by regulating ATG3 (a regulatory component of autophagosome) and BECLIN-1 (a key protein involved in the autophagosome formation) [20, 42]. As for OV, a study from Ma and colleagues claimed that genetic variants of CASP8 could protect against carcinogenesis and delay tumor onset [21]. FOXO1, as a vital member of the mammalian Forkhead Box protein (FOXOs) family, is essential in various intra-cellular functions, including autophagy [9]. Liu and colleagues investigated FOXO1 expression in OV patients and reported that FOXO1 was an independent prognostic biomarker in OV [43], which was consistent with our findings. Ma and colleagues demonstrated that the phosphorylation of FOXO1 regulated by ITGA2 could regulate resistance to paclitaxel in OV [19]. Additionally, in our study, we initially explored the role of FOXO1 in tumorigenesis and progression of OV. The results indicated that FOXO1 significantly promoted tumor invasiveness, migration, and proliferation in OV cell lines, though the underlying mechanism needs further investigation.

However, there remained several limitations in our research. Firstly, the autophagy-related signature should be further validated in a large database, in order to promote clinical application and improve OV prognosis. Though we verified the expression of FOXO1 in tissues and the importance of FOXO1 in OV progression, the underlying biological functions need further investigation. Considering the significant relationship between immune microenvironment and the autophagy-related signature, the immune signature might add value to the autophagy-related signature, which should be further evaluated.

## Conclusion

Briefly, we identified and validated an autophagy-related signature (including FOXO1 and CASP8) to evaluate prognosis, predict therapy response, and guide clinical treatment in OV. Comprehensive analysis identified significant relationships between the autophagy patterns and immune cell infiltration, which hinted individual decision-making. Especially, our findings identified the role of FOXO1 in OV metastasis and presented a potential therapeutic target for highly-malignant OV, though the underlying mechanism still needs further investigation.

## Supplementary Information

**Additional file 1: Supplement figure 1.** The clinical characteristics of OV patients, stratified by the autophagy- related 2-gene signature. (A-D) The stacked bar plots presented the distribution of various clinical features, including age, race, grade, and clinical FIGO (the International Federation of Gynecology and Obstetrics) stage, among low-risk and high-risk groups. (E) The Sankey diagram indicated the distribution of each OV patient, based on the autophagy-related signature and clinical features, including age, pathological grade, and clinical FIGO stage. Rows represented feature variables, while lines represent the distribution of the same sample in various feature variables. NS, no significance.

## Data Availability

The data that support the findings of this research are available from the corresponding author upon reasonable requests.
